# Di-μ-nitrosyl-bis­[(η^5^-penta­methyl­cyclo­penta­dien­yl)ruthenium(0)](*Ru—Ru*). Corrigendum

**DOI:** 10.1107/S1600536810011980

**Published:** 2010-04-14

**Authors:** Matthew Pearsal, Milan Gembicky, Paulina Dominiak, Anna Larsen, Philip Coppens

**Affiliations:** a Department of Chemistry, CNS 359, Ithaca College, Ithaca, NY 14850, USA; b Department of Chemistry, State University of New York at Buffalo, 732 NS Complex, Buffalo, NY 14260, USA

## Abstract

The acknowledgement in the paper by Pearsal, Gembicky, Dominiak, Larsen & Coppens [*Acta Cryst.* (2007), E**63**, m2596] is extended and an omitted reference is added.

## Abstract 2

Corrigendum to *Acta Cryst.* (2007), E**63**, m2596.

In the paper by Pearsal *et al.* (2007) ▶, the complete acknow­ledgement should read: "Support of the REU program CHE-0453206 by the National Science Foundation is gratefully acknowledged. The synthetic route to the title compound in this work (*via* 2-propanol oxidation) was first developed in the context of ALs PhD dissertation completed in 1996 under the supervision of Dr John L. Hubbard, whose contribution to this project is gratefully acknowledged."A reference for the PhD dissertation [Larsen (née Svet­lan­ova), 1996 ▶] is also added.
